# Disengagement from care in a decentralised primary health care antiretroviral treatment programme: cohort study in rural South Africa

**DOI:** 10.1111/tmi.12135

**Published:** 2013-06-03

**Authors:** Portia C Mutevedzi, Richard J Lessells, Marie-Louise Newell

**Affiliations:** 1 Africa Centre for Health and Population Studies, University of KwaZulu-NatalSomkhele, South Africa; 2 Department of Infection and Population Health, University College LondonLondon, UK; 3 Department of Clinical Research, London School of Hygiene and Tropical MedicineLondon, UK; 4 Institute of Child Health, University College LondonLondon, UK

**Keywords:** HIV-1, antiretroviral agents, primary health care, delivery of health care, lost to follow-up, disengagement from care

## Abstract

**Objective**To determine rates of, and factors associated with, disengagement from care in a decentralised antiretroviral programme.

**Methods**Adults (≥16 years) who initiated antiretroviral therapy (ART) in the Hlabisa HIV Treatment and Care Programme August 2004–March 2011 were included. Disengagement from care was defined as no clinic visit for 180 days, after adjustment for mortality. Cumulative incidence functions for disengagement from care, stratified by year of ART initiation, were obtained; competing-risks regression was used to explore factors associated with disengagement from care.

**Results**A total of 4,674 individuals (median age 34 years, 29% male) contributed 13 610 person-years of follow-up. After adjustment for mortality, incidence of disengagement from care was 3.4 per 100 person-years (95% confidence interval (CI) 3.1–3.8). Estimated retention at 5 years was 61%. The risk of disengagement from care increased with each calendar year of ART initiation (*P* for trend <0.001). There was a strong association between disengagement from care and higher baseline CD4+ cell count (subhazard ratio (SHR) 1.94 (*P* < 0.001) and 2.35 (*P* < 0.001) for CD4+ cell count 150–200 cells/μl and >200 cells/μl respectively, compared with CD4 count <50 cells/μl). Of those disengaged from care with known outcomes, the majority (206/303, 68.0%) remained resident within the local community.

**Conclusions**Increasing disengagement from care threatens to limit the population impact of expanded antiretroviral coverage. The influence of both individual and programmatic factors suggests that alternative service delivery strategies will be required to achieve high rates of long-term retention.

## Introduction

Long-term retention in human immunodeficiency virus (HIV) care is one of the key challenges facing public health systems in low-income and middle-income countries (Baggaley *et al*. 2012; World Health Organization 2012). This is especially true in South Africa, which has rapidly expanded access to antiretroviral therapy (ART) through the public health system since 2004 and now operates the largest ART programme in the world (Joint United Nations Programme on HIV/AIDS (UNAIDS) (2012). The South African National Strategic Plan 2012–2016 has a target of 80% overall retention at 5 years (Department of Health 2011), yet there are few published data on long-term retention. Here, we utilise linked longitudinal data from a rural HIV treatment programme and a demographic surveillance system to quantify rates of disengagement from care, after adjustment of loss to follow-up (LTF) for mortality, to explore the association of disengagement from care with key individual, household and health system characteristics and finally to determine residency status for those disengaged from care.

## Methods

### Hlabisa HIV Treatment and Care Programme and Africa Centre Demographic Information System (ACDIS)

The Hlabisa HIV Treatment & Care Programme (HHTCP) delivers comprehensive HIV treatment and care in Hlabisa health subdistrict, northern KwaZulu-Natal (Mutevedzi *et al*. 2010; Houlihan *et al*. 2011). Treatment and care is devolved to 17 primary health care (PHC) clinics and is delivered largely by nurses and counsellors in adherence to national guidelines. All treatment and care is provided free of charge. Clinical information is collected at time of ART initiation for all individuals and is transferred from standardised paper-based clinic records to a Microsoft® SQL database housed at the Africa Centre. Subsequent visits on ART and changes in vital status (death, LTF, transfer out) are captured on paper-based records and transferred to the database each month.

Since 2000, demographic data have been collected from around 11 000 households and 85 000 individuals in an area of 438 km^2^ situated within Hlabisa health subdistrict (Tanser *et al*. 2008). An estimated 30–40% of people in the HHTCP live in this area; six of the 17 PHC clinics are situated within the surveillance area.

Deaths of individuals classified as lost to follow-up from the programme were identified through linkage of individual records between programme and surveillance databases; in October 2011, a separate exercise was undertaken, wherein additional deaths were identified through linkage with the National Population Register (NPR).

### Data analysis

This analysis included all adults (≥16 years) who were members of the demographic surveillance system and initiated ART in the HHTCP between 1 August 2004 and 31 March 2011. In-migration prior to ART initiation was defined as migration into the area in the 12 months before the date of ART initiation. LTF was defined as 180 days having elapsed since the last recorded clinic visit, in adherence to established definitions (Chi *et al*. 2011). Disengagement from care was defined as LTF adjusted for mortality: individuals recorded as LTF who were found, through linkage, to have died within 180 days of the last recorded clinic visit were reclassified as deaths. Individuals who formally transferred care out of the programme to another health facility were defined as transfers out (TRO).

Observation started at date of ART initiation, or date of transfer into programme for those already on ART, and ended at date of death, date of last clinic visit (for LTF and TRO) or database closure (12 July 2012). Cumulative incidence functions for disengagement from care, stratified by year of ART initiation and adjusted for all covariates in the final multivariable model, were obtained using the Coviello and Boggess method (Coviello & Boggess 2004). Overall programme retention at 5 years was estimated from the cumulative incidence curve. As disengagement from care occurs in the context of competing risks of mortality and transfers, competing-risks regression was used to explore factors associated with disengagement (Bakoyannis & Touloumi 2008; Gutierrez 2010). Individual and household socio-demographic characteristics, as well as geographical factors, were obtained from ACDIS with reference to the last clinic visit date. The subhazard ratio (SHR) was derived as the risk of disengagement for individuals in a certain category compared with a reference category, in the presence of competing risks of death and transfer out. All analyses were conducted using STATA 11.2 (StataCorp, College Station, Texas), and all results are reported at 5% significance level.

### Ethics statement

Ethical approval was granted by the Biomedical Research Ethics Committee of the University of KwaZulu-Natal (refs. BE066/07 & E134/06) and the Health Research Committee of the KwaZulu-Natal Department of Health (refs. HRKM012/07 & HRKM032/08).

## Results

### Cohort characteristics

A total of 4674 adult members of the demographic surveillance system initiated ART between 1 August 2004 and 31 March 2011 (Table 1) and contributed 13 610 person-years of follow-up (median 2.77 years).

**Table 1:** Characteristics of 4674 adult members of the demographic surveillance who initiated antiretroviral therapy (ART)

	*n*	% (median)	95% CI (IQR)
Sex, male	1354	29.0	27.7–30.3
Age, years	4674	(34)	(28–42)
Baseline CD4+ cell count*, cells/μl	4477	(129)	(67–182)
Year of ART initiation			
2004–2006	760	16.3	15.2–17.4
2007	758	16.2	15.2–17.3
2008	1009	21.6	20.4–22.8
2009	882	18.9	17.8–20.2
2010	981	21.0	19.9–22.2
2011†	284	6.1	5.4–6.8
Current clinic‡			
Township	1648	35.3	33.9–36.6
Town	663	14.2	13.2–15.2
Hospital	182	3.9	3.3–4.5
Rural clinics (×14)	2181	46.7	45.2–48.1
Ever switched clinic			
No	3432	87.6	86.6–88.7
Yes	372	9.5	8.6–10.4
Unknown	112	2.9	2.3–3.4
Transfer into the programme	160	3.4	2.9–3.9
In-migration prior to ART initiation	499	10.7	9.8–11.6
Number of days from eligibility to initiation	4410	(49)	(30–78)
Employed at initiation			
No	1683	36.0	34.6–37.4
Yes	2361	50.5	49.1–52.0
Missing	630	13.5	12.5–14.5
Employed at last follow-up			
No	2732	58.5	57.0–60.0
Yes	1650	35.3	33.9–36.7
Missing	292	6.3	5.6–6.9
Education (highest level achieved)			
Secondary	2195	47.0	45.5–48.4
Primary	894	19.1	18.0–20.3
None	1203	25.7	24.5–27.0
Missing	382	8.2	7.4–9.0
Distance to nearest clinic, km			
Median	3303	(2.3)	(0.8–9.8)
>5 km	1278	38.7	37.0–40.4
Distance to nearest main tarred road (km)			
Median	3303	(2.3)	(0.8–9.8)
>5 km	1278	27.3	26.1–28.6
Marital status			
Married	451	9.7	8.8–10.5
Never married	3580	76.8	75.6–78.0
Engaged	167	3.6	3.1–4.1
Divorced/widowed/separated	463	9.9	9.1–10.8
Household owner			
Self	1470	37.4	35.9–38.9
Spouse	244	6.2	5.4–7.0
Other	2220	56.4	54.9–58.0
Gender of household owner, male	2059	54.0	52.5–55.6
Age of household owner	3810	(50)	(41–60)
Number of individuals in household	3859	(8)	(5–12)

CI, confidence interval; IQR, interquartile range.

Figures in parentheses represent medians and interquartile ranges.

* Baseline CD4+ cell count defined as the last measured CD4+ cell count up to 180 days prior to date of ART initiation.

† Includes to 31 March 2011.

‡ Although only six clinics are situated within the demographic surveillance area, some members of the surveillance access clinics outside the surveillance area but within the subdistrict.

### Disengagement from care

**Figure 1:**
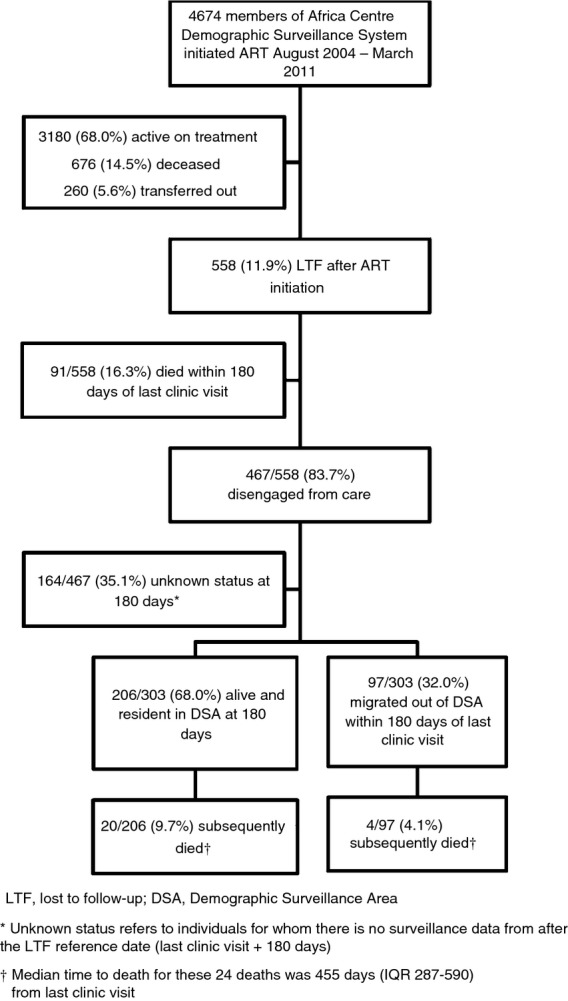
Flow diagram of 4674 individuals from time of ART initiation to determination of vital status after disengagement from care.

**Figure 2:**
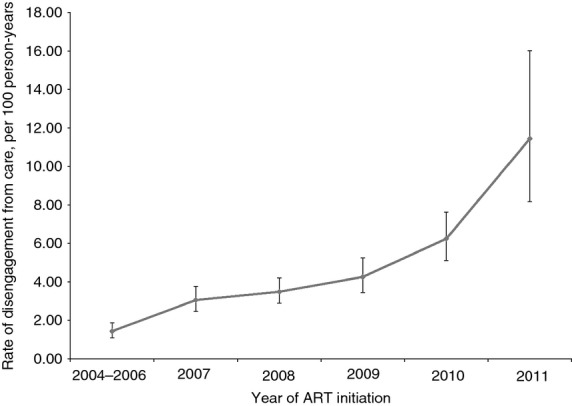
Rates of disengagement from care, stratified by calendar year of antiretroviral therapy initiation.

Overall, 676 (14.5%) died, 260 (5.6%) transferred out of the programme, 558 (11.9%) were LTF, and 3180 (68.0%) were alive on ART (Figure 1). Of those LTF, 91 (16.3%) had died within 180 days of their last clinic visit [median time 7 days, interquartile range (IQR) 0–36]. Consequently, the true mortality was 16.4% (*n* = 767) and the proportion disengaged from care was 10.0% (*n* = 467); the rate of disengagement was 3.4 per 100 person-years [95% confidence interval (CI) 3.1–3.8]. After adjustment for mortality, the first 3 months post-ART initiation remained the period with the highest rate of disengagement (Table 2). The rate of disengagement increased for each calendar year of ART initiation (Figure 2). Overall programme retention at 5 years was estimated at 61%.

**Table 2:** Rates of loss to follow-up (LTF) and disengagement from care (after adjustment of LTF for mortality), per 100 person-years

		Loss to follow-up			Disengagement from care		
Cohort period (months)	Person-time	Failures	Rate	95% CI	Failures	Rate	95% CI
0–3	1113.62	91	8.17	6.65–10.04	58	5.21	4.03–6.74
3–12	3076.44	121	3.93	3.29–4.70	99	3.22	2.64–3.92
12–24	3486.20	127	3.64	3.06–4.33	112	3.21	2.67–3.87
24–36	2538.28	97	3.82	3.13–4.66	86	3.39	2.74–4.19
36	3395.74	122	3.59	3.01–4.29	112	3.30	2.74–3.97
Total	13610.28	558	4.10	3.77–4.45	467	3.43	3.13–3.76

CI, confidence interval.

### Factors associated with disengagement from care

**Figure 3:**
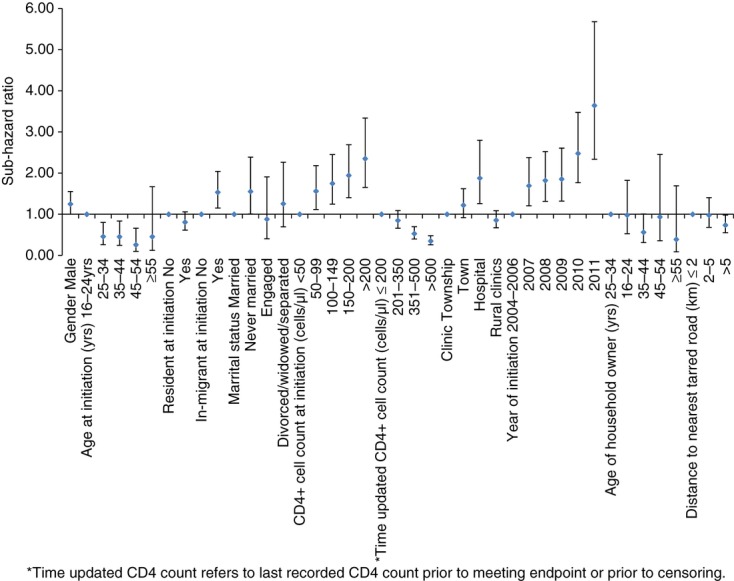
Subhazard ratios for disengagement from care in the presence of competing risks of mortality and transfer out. Model adjusted for education level and employment as at time of last clinic visit.

There was a significant trend in increasing risk of disengagement from care with increasing baseline CD4+ cell count: SHR 1.56, 1.75, 1.94 and 2.35 for CD4+ cell count 50–99, 100–149, 150–200 and >200 cells/μl, respectively, compared with CD4 count <50 cells/μl (Figure 3). Year of ART initiation was strongly associated with disengagement from care, with risk progressively increasing with each calendar year (*P* < 0.002 across all years). Individuals who had migrated into the AC surveillance area in the year prior to ART initiation were 53% more likely to disengage from care compared with those resident throughout (SHR 1.53, *P* = 0.03).

### Outcomes after disengagement from care

Of the 467 individuals disengaged from care, most (303, 65.9%) had surveillance data available beyond 180 days from last clinic visit. The majority (206/303, 68.0%) remained resident within the surveillance area, whereas 97 (32.0%) had migrated outside the surveillance area (median time from last clinic visit to migration 2 days, IQR 2–157). Overall, 24 individuals died subsequent to disengagement, a median of 455 days (IQR 287–590) after their last clinic visit.

## Discussion

In South Africa, the rapid scale-up of HIV treatment programmes has put enormous strain on health systems, especially in rural areas, where health systems remain critically under-resourced (Versteeg & Couper 2011). The programme reported here has rapidly expanded ART coverage resulting in positive impacts on population-level mortality and on risk of HIV acquisition (Herbst *et al*. 2009; Cooke *et al*. 2010; Mutevedzi *et al*. 2010; Bor *et al*. 2013; Tanser *et al*. 2013). Increasing disengagement from care has the potential to limit these gains, and these data highlight the need to develop successful long-term retention strategies.

Overall programme retention at 5 years was estimated at 61%, below the target of 80% in the National Strategic Plan (Department of Health 2011). There are limited data on long-term retention, and this is the first report with long-term follow-up from a rural PHC programme. A combined analysis of eight urban public sector ART programmes reported overall programme retention of 64% at 3 years (Cornell *et al*. 2010), whereas two individual programmes reported combined LTF and mortality at 5 and 6 years, respectively, of 35.3% and 37.4% (Boulle *et al*. 2010; Nglazi *et al*. 2011). Our estimated retention at 5 years of 61% is broadly comparable to these studies, suggesting there may be relatively limited heterogeneity in programmatic performance.

The finding that disengagement from care has increased with each year of programme scale-up is of major concern: individuals enrolled on ART in 2010 and 2011 had more than double the risk of disengagement compared with those enrolled in 2004–2006. This supports findings from other programmes (Boulle *et al*. 2010; Fatti *et al*. 2011; Nglazi *et al*. 2011), although only one had corrected LTF for mortality (Boulle *et al*. 2010). This may reflect health systems struggling to cope with increasing patient load. In this programme, service delivery has been devolved to PHC level since inception in 2004, so whether models of care with further decentralisation outside the health system into the community could improve long-term retention should be evaluated (Decroo *et al*. 2011; Fatti *et al*. 2012; Rich *et al*. 2012).

With moves towards earlier initiation of ART and the expanded use of treatment for prevention, concern has been raised about whether adherence and retention will be different in those who are less symptomatic at the time of ART initiation (Lockman & Sax 2012). In this light, the association between higher pre-treatment CD4+ cell count and increasing disengagement is of concern. Mathematical models exploring HIV treatment for prevention have demonstrated that the impact on HIV incidence is particularly sensitive to changes in programme retention but have assumed low rates of disengagement, based on data from the early phase of treatment scale-up, which may not reflect the current situation (Granich *et al*. 2009; Hontelez *et al*. 2011; Eaton *et al*. 2012).

There are high levels of population mobility in this rural area, and migration dynamics are complex (Muhwava *et al*. 2010). About one in ten people who initiated ART in this programme had migrated into the area in the year before ART initiation, consistent with our previous findings that people return to the family home when unwell and to access ART (Welaga *et al*. 2009; Olgiati *et al*. 2012). These in-migrants were more likely to disengage after ART initiation, which may partially reflect people returning to employment (Bor *et al*. 2012). The highest rates of disengagement from care were observed in the first 3 months after ART initiation. Disengagement during this early time period could also be explained by early morbidity, drug toxicity or problems with the patient/provider relationship at the healthcare facility. These early losses, combined with early mortality, highlight that the first few months after ART initiation remain a critical period when more intensive support might be required, and it may be that programmes are increasingly unable to provide that extra support.

Interpretation of these results should be subject to some limitations. The reclassification of deaths included those who died within 180 days of last clinic visit. This is a relatively long time period and therefore might encompass some people who disengaged from care and died as a result of ART interruption, thus underestimating true disengagement from care and its impact. However, the median time to death for those reclassified was 7 days and almost three-quarters died within a month of the last clinic visit, suggesting that most deaths occurred before the next scheduled clinic visit. We were unable to identify individuals that disengaged but then reengaged with care, an occurrence that has been reported to be quite frequent elsewhere (Kranzer *et al*. 2010). Failure to capture these interruptions in care could underestimate the potential impact of disengagement on both individual- and population-level outcomes. The additional support provided by a non-governmental organisation might contribute to differences in programmatic outcomes, so this cannot necessarily be taken to be truly representative of all public sector programmes.

In summary, increasing rates of disengagement from care underline the need for new service delivery strategies to achieve the high rates of long-term retention needed to sustain the positive impacts of ART roll-out.
